# Supplementary material for: Transcriptome analysis identifies CCR7 and cell adhesion molecules as mediators of B cell migration to the bursa of Fabricius during chicken embryonic development
Source: BMC Genomics. 2025 Jun 3;26:555. doi: 10.1186/s12864-025-11749-w (PMC12131466; doi:10.1186/s12864-025-11749-w)
Supplement: Supplementary file 1 — Supplementary Material 1 [file 12864_2025_11749_MOESM1_ESM.pdf]

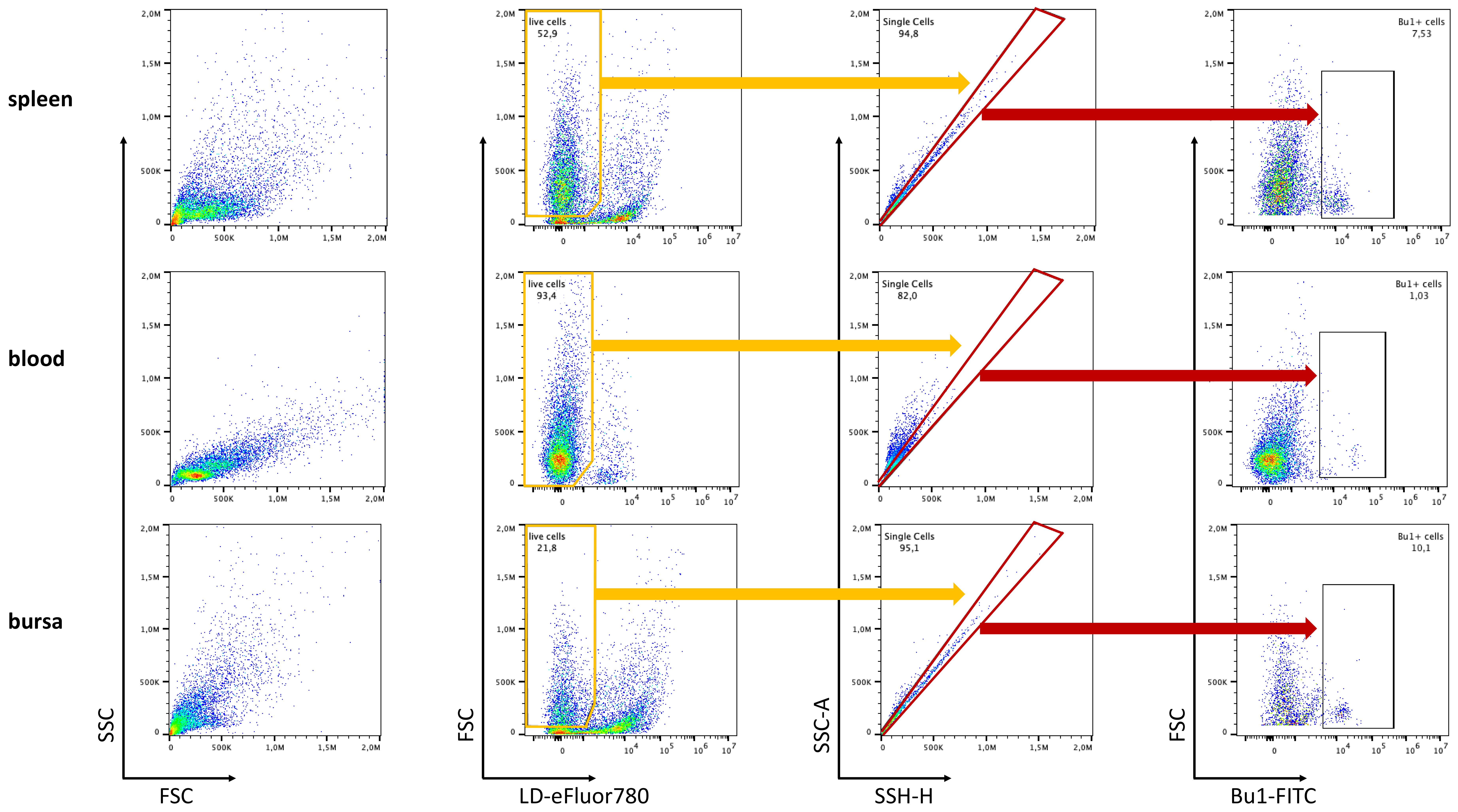

Fig. S1: Schematic representation of the flow-cytometry gating strategy for B cell percentages in the different tissues. Flow-cytometry analysis of B cells (Bu1-FITC^{+}) within single live cells (LD-eFluor780^{-}) in percentages from the spleen (top), blood (middle), and bursa (bottom).

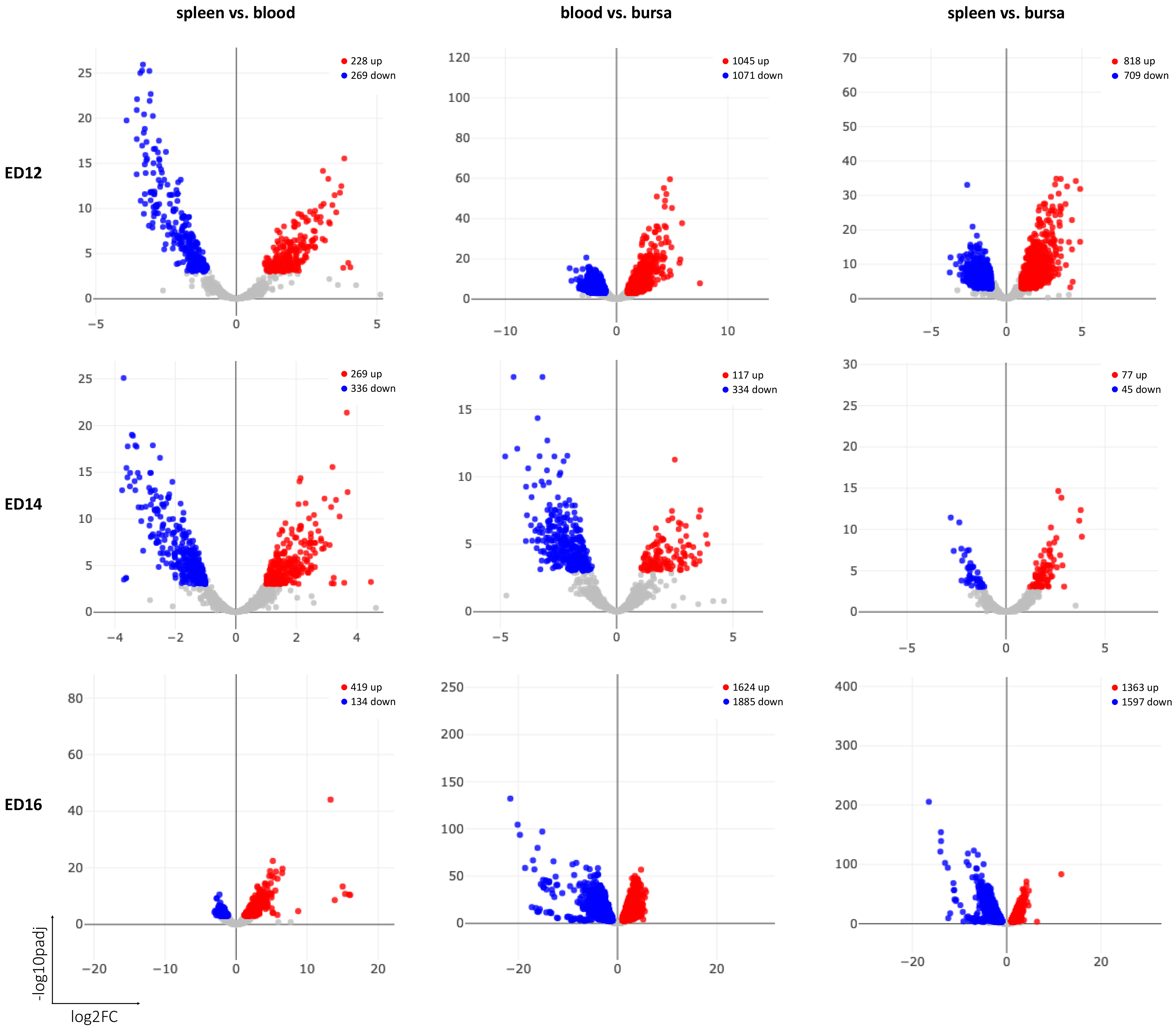

Fig. S2: Volcano plots of the up- and downregulated DEGs between the spleen vs. bursa (left), blood vs. bursa (middle), and spleen vs. bursa (right) on ED12 (top), ED14 (middle), and ED16 (bottom). The $log_2(FC)$ reflects the mean fold-change (FC) in expression levels for each gene, and each dot represents one gene. DEGs are defined as $p_{adjust} < 0.001$, FC > |2|. DEGs with higher expression (i.e., higher in the spleen than in blood) are represented by red dots, while DEGs with lower expression (i.e., lower in the spleen than in blood) are denoted by blue dots. Genes with no substantial change in expression are indicated as gray dots.

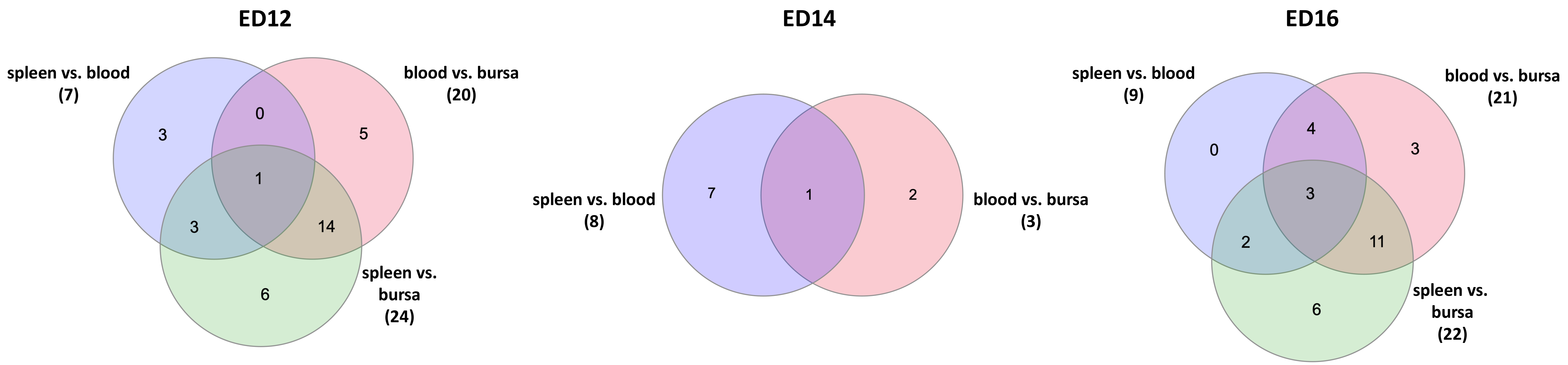

Fig. S3: Venn diagram of shared DEGs associated to the GO-term locomotion or cell adhesion molecules between the tissues spleen vs. blood (blue), spleen vs. bursa (green), blood vs. bursa (red) at ED12 (top), ED14 (middle) and ED16 (bottom).

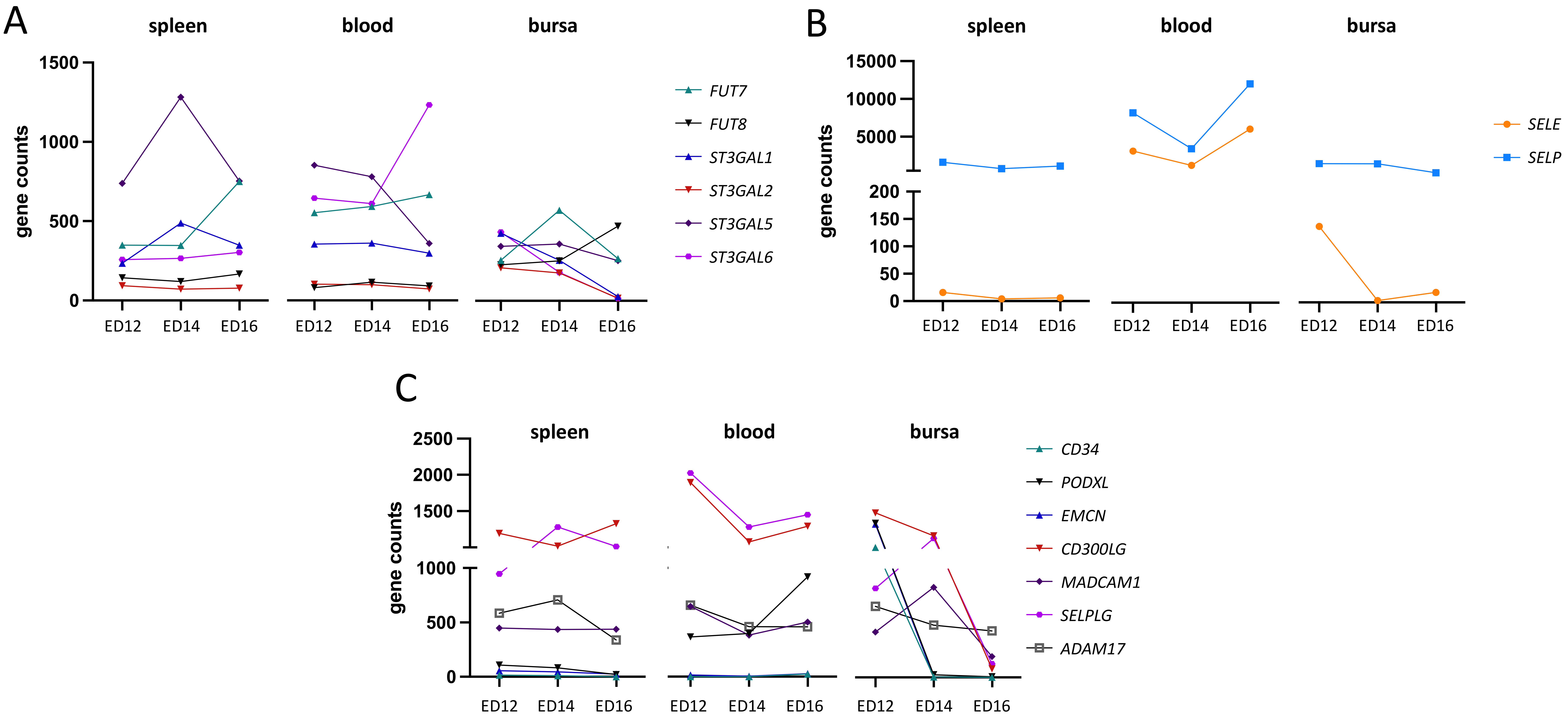

Fig. S4: Gene expression profiles of the (A) fructosyl- and sialyl transferases (B) selectin E and P and (C) Selectin L ligands over time (ED12, ED14, and ED16) in the spleen (left), blood (middle), and bursa (right). Each symbol and color indicates one individual gene, data are cumulative from 3 experiments (*n*=3 per B cell sample and time-point) and represented as mean.

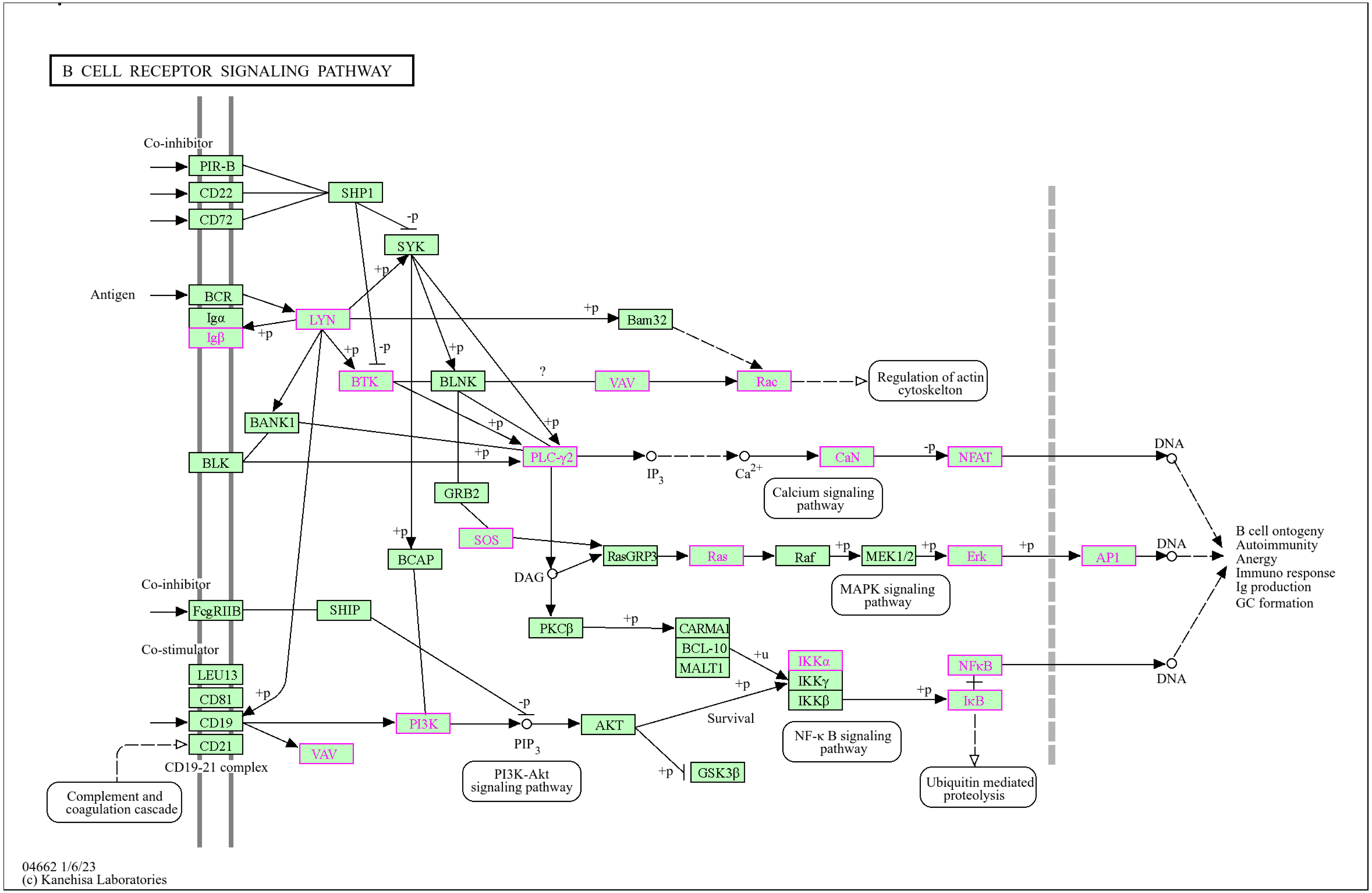

Fig. S5: KEGG pathway map for human B cell receptor signaling pathway. DEGs associated with the GO term B cell activation (Tab. S4) in the bursa at least at one time point comparison and mapped to the B cell receptor signaling pathway are marked in pink.

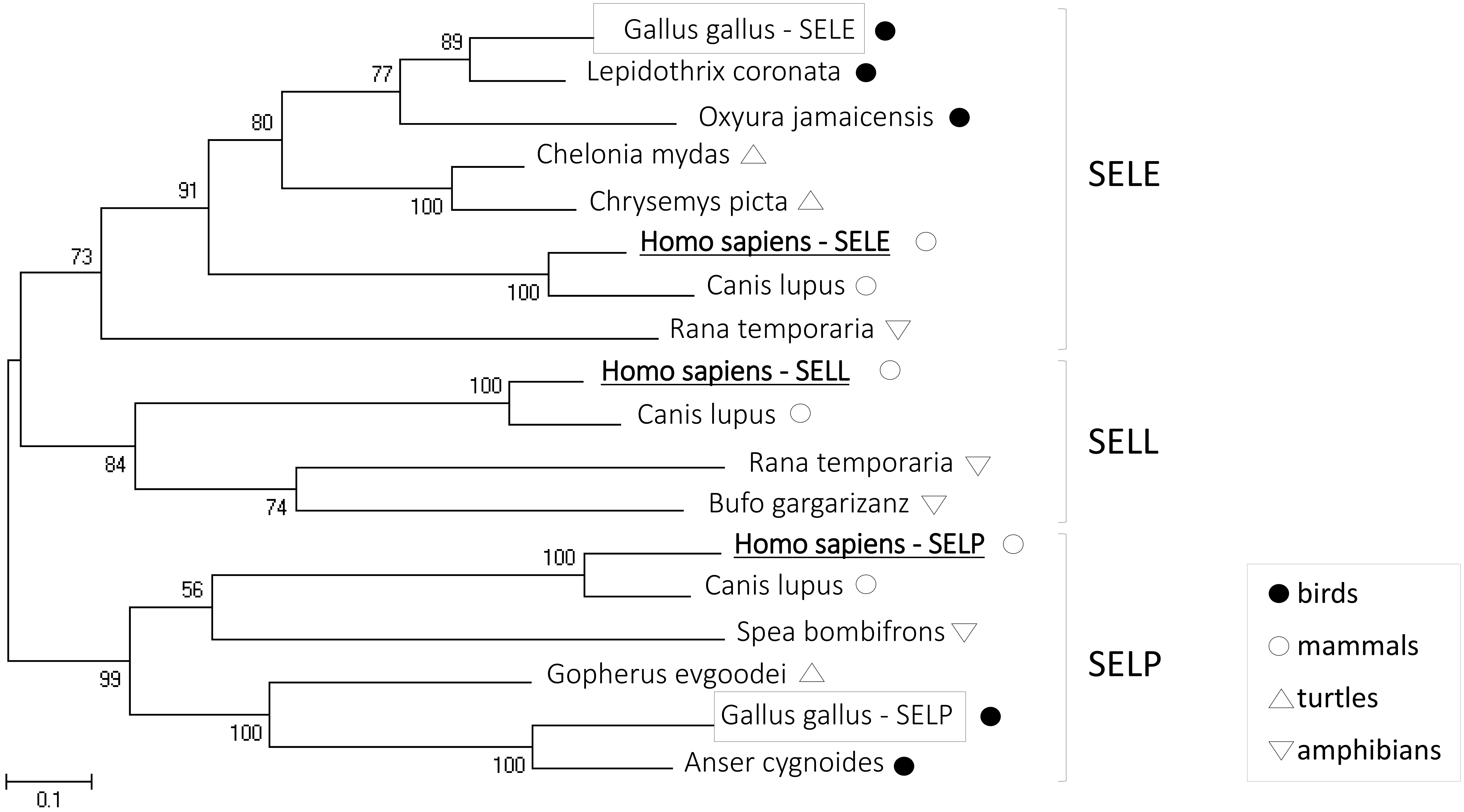

Fig. S6: Phylogenetic relationship of chicken selectins with other vertebrate orthologs. Both chicken selectins are highligted by grey boxes. Human E-selectin (SELE), L-selectin (SELL) and P-selectin (SELP) are shown in bold and underlined. Maximum likelihood tree was generated in MEGA6.06 software. Bootstrap support values (out of 100) are shown at each node. Scale bar shows number of amino acid substitutions per site. The following sequences were used to generate the phylogenetic tree: human SELL (accession number NP000646), human SELE (NP000441), human SELP (NP002996), *G. gallus* SELE (XP046754151), *G. gallus* SELP (XP422207), and selectin genes from various vertebrate species *Canis lupus* (XP025286208, XP025286205, XP025286209), *Rana temporaria* (XP040215647, XP040215645), *Bufo gargarizans* (XP044156813), *Spea bombifrons* (XP053326045), *Chelonia mydas* (XP037762768), *Gopherus evgoodei* (XP030427921), *Anser cygnoides* (X066857252), *Lepidothrix coronata* (XP017671135), *Oxyura jamaicensis* (XP035189260), *Chrysemys picta* (XP_065410664).

Tab. S1: Shared DEGs associated to the GO terms locomotion and cell adhesion molecules (n=37) between the spleen vs. blood, blood vs. bursa and spleen vs. bursa on ED12, ED14 and ED16

**ED12**		**ED14**		**ED16**	
shared between all: spleen vs. blood, blood vs. bursa and spleen vs. bursa	NET1	shared between all: spleen vs. blood, blood vs. bursa and spleen vs. bursa		shared between all: spleen vs. blood, blood vs. bursa and spleen vs. bursa	PLXNC1<br>ITGB1<br>JAM3
shared between spleen vs. blood and blood vs. bursa		shared between spleen vs. blood and blood vs. bursa	ITGB2	shared between spleen vs. blood and blood vs. bursa	BSG<br>RAC2<br>ITGAV<br>ITGA6
shared between spleen vs. blood and spleen vs. bursa	ITGB1<br>ITGAV<br>MCAM	shared between spleen vs. blood and spleen vs. bursa		shared between spleen vs. blood and spleen vs. bursa	CNTN1<br>CXCR4
shared between blood vs. bursa and spleen vs. bursa	EPHB6<br>CYFIP1<br>TRIO<br>BSG<br>KIF5B<br>PIKFYVE<br>SEMA7A<br>RHOH<br>ELF1<br>PLXNA1<br>FAR2<br>ANTXR2<br>THSD7B<br>SGCE	shared between blood vs. bursa and spleen vs. bursa		shared between blood vs. bursa and spleen vs. bursa	CCR7<br>NOTCH1<br>UNC5B<br>CX3CR1<br>CYFIP1<br>CYFIP2<br>SEMA4B<br>ATRN<br>MADCAM1<br>PCDH12<br>ITGB2

Tab. S2: Protein-protein interaction (PPI) network analysis represents the associated GO term, FDR and p-value, number of background genes and genes included in the network

term name	description	FDR value	# background genes	# genes in network	p-value
GO:0009986	Cell surface	1.68E-10	499	15	$1.26E^{-13}$
GO:0005886	Plasma membrane	3.2E-8	3837	28	$4.79E^{-11}$
GO:0031224	Intrinsic component of membrane	4.13E-8	4295	29	$9.28E^{-11}$
GO:0098636	Protein complex involved in cell adhesion	7.69E-8	32	6	$2.31E^{-10}$

Tab. S3: List of genes identified in the gene set ´cell surface´ in the comparisons spleen vs. bursa and blood vs. bursa

**cell surface**			
spleen vs. bursa	ABCB11<br>ADAMTS7<br>ANXA2<br>APP<br>B2M<br>BMPR2<br>BTN1A1<br>C1QBP<br>CD83<br>CD8A<br>CD99L2<br>CIITA<br>CLIC4<br>CLSTN1<br>CTNND1<br>CTSB<br>CTSZ<br>DCBLD2<br>ELP<br>FCER1G<br>GLRA1<br>GOT2<br>HMMR<br>HSP90AA1<br>HSP90AB1<br>HSPA8<br>HSPD1<br>IL15RA<br>ITGB1<br>ITGB2<br>JAM3<br>KCNH1<br>LBP<br>LRP8<br>LRRC8A<br>LYPLA1<br>MFGE8<br>NRROS<br>PDGFB<br>PECAM1<br>PRNP<br>S1PR1<br>SCNN1B<br>SULF2<br>TGFB1<br>TIMP2<br>TLR2<br>TNFRSF10B<br>TNFRSF1A<br>TSPAN32<br>VSIR<br>VTCN1	blood vs. bursa	ADAMTS7<br>ANXA2<br>ANXA4<br>ANXA5<br>APP<br>BTN1A1<br>CD44<br>CKAP4<br>CLU<br>CTSB<br>CXCR4<br>EPHB6<br>FAS<br>FLT3<br>GOT2<br>HMMR<br>HSP90AA1<br>HSP90AB1<br>IL2RG<br>ITGB1<br>JAM3<br>KIT<br>LBP<br>LRRC8A<br>LYPLA1<br>MFGE8<br>MSN<br>NRROS<br>NTSR1<br>P2RX7<br>PECAM1<br>PRNP<br>S100A10<br>SCUBE1<br>SLC9A1<br>TIMP2<br>TLR2<br>TNFRSF1A

Tab. S4: List of DEGs identified between the different time-points in the bursa associated with the GO term B cell activation

**B cell activation**					
ED12 vs. ED14	GRAPL<br>HRAS<br>ITPR1<br>JUN<br>NFKB1<br>NFKB2<br>NFKBIA<br>PIK3CG<br>PRKCD	ED14 vs. ED16	CD79B<br>CHUK<br>GRAPL<br>NFKBIA<br>PIK3CG<br>VAV3	ED12 vs. ED16	BTK<br>CHUK<br>HRAS<br>JUN<br>LOC107050724<br>LYN<br>MAPK3<br>MAPK8<br>MAPK9<br>NFATC2<br>NFATC3<br>NFKB2<br>PIK3CD<br>PIK3CG<br>PLCG2<br>PPP3CA<br>PRKCD<br>PTPRC<br>RAC1<br>SOS2

Tab. S5: qRT-PCR primers

name	Sequence 5´-> 3´	Amplicon size (nt)	orientation	Tm°C
CCR7_fw	TGTGCTGGGAACAACGTCACCG	107	FWD	61,9
CCR7_rv	GCAGCACGGAAATCACGGACCT		REV	61,8
PECAM1_fw	CCAGTGGCATGAAAACTAGCAAGGT	76	FWD	59,5
PECAM1_rv	ACACTGCCCAAGCTGGTGTTCC		REV	61,6
S1PR1_fw	GTTGGCTGGCGTGGCTTACACT	95	FWD	62
S1PR1_rv	TGCTGCCCTCCCTTACGAACCA		REV	61,9
ITGB1_fw	CATGCATGGCAGGGAACGGACA	135	FWD	61,8
ITGB1_rv	GCTCTGCACAGACACCAAGGCA		REV	61,6
